# *N* ^1^,*N* ^3^-Bis(pyridin-3-ylmeth­yl)isophthalamide dihydrate

**DOI:** 10.1107/S1600536811031965

**Published:** 2011-08-17

**Authors:** Ying-ying Dong, Xi-chuan Cao

**Affiliations:** a School of Materials Science and Engineering, China University of Mining and Technology, Xuzhou, Jiangsu Province 221008, People’s Republic of China

## Abstract

The complete organic molecule in the title dihydrate, C_20_H_22_N_4_O_4_, is generated by crystallographic twofold symmetry, with two C atoms lying on the rotation axis. The symmetry unique pyridine ring forms a dihedral angle of 83.16 (8)° with the central benzene ring. In the crystal, inter­molecular N—H⋯O, O—H⋯N and O—H⋯O hydrogen bonds connect the components into a two-dimensional network lying parallel to (101).

## Related literature

For information on amide derivatives used in the construction of metal-organic frameworks, see: Luo *et al.* (2007 ▶, 2009 ▶). 
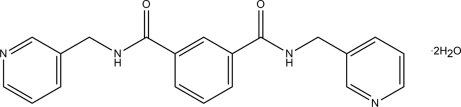


## Experimental

### 

#### Crystal data

C_20_H_18_N_4_O_2_·2H_2_O
*M* *_r_* = 382.42
Monoclinic, 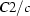

*a* = 23.0097 (8) Å
*b* = 7.0040 (2) Å
*c* = 12.4483 (4) Å
β = 107.493 (2)°
*V* = 1913.39 (11) Å^3^
*Z* = 4
Mo *K*α radiation
μ = 0.10 mm^−1^
*T* = 296 K
0.20 × 0.20 × 0.15 mm

#### Data collection

Bruker SMART CCD diffractometer
Absorption correction: multi-scan (SADADS; Sheldrick, 1996 ▶) *T*_min_ = 0.981, *T*_max_ = 0.986
7105 measured reflections
1687 independent reflections
1350 reflections with *I* > 2σ(*I*)
*R*_int_ = 0.026

#### Refinement

*R*[*F* ^2^ > 2σ(*F* ^2^)] = 0.039
*wR*(*F* ^2^) = 0.113
*S* = 1.02
1687 reflections
137 parameters
H atoms treated by a mixture of independent and constrained refinement
Δρ_max_ = 0.17 e Å^−3^
Δρ_min_ = −0.14 e Å^−3^

### 

Data collection: *SMART* (Bruker, 1998 ▶); cell refinement: *SAINT* (Bruker, 1998 ▶); data reduction: *SAINT*; program(s) used to solve structure: *SHELXS97* (Sheldrick, 2008 ▶); program(s) used to refine structure: *SHELXL97* (Sheldrick, 2008 ▶); molecular graphics: *DIAMOND* (Brandenburg, 1999 ▶); software used to prepare material for publication: *SHELXTL* (Sheldrick, 2008 ▶).

## Supplementary Material

Crystal structure: contains datablock(s) global, I. DOI: 10.1107/S1600536811031965/lh5294sup1.cif

Structure factors: contains datablock(s) I. DOI: 10.1107/S1600536811031965/lh5294Isup2.hkl

Supplementary material file. DOI: 10.1107/S1600536811031965/lh5294Isup3.cml

Additional supplementary materials:  crystallographic information; 3D view; checkCIF report

## Figures and Tables

**Table 1:** Hydrogen-bond geometry (Å, °)

*D*—H⋯*A*	*D*—H	H⋯*A*	*D*⋯*A*	*D*—H⋯*A*
N1—H1*A*⋯O2^i^	0.86	2.05	2.859 (2)	156
O2—H2*W*⋯O1^ii^	0.95 (3)	1.94 (3)	2.875 (2)	169 (3)
O2—H1*W*⋯N2	0.95 (3)	1.90 (3)	2.849 (2)	178 (3)

Symmetry codes: (i) 
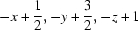
; (ii) 
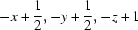
.

**Fig. 1.**
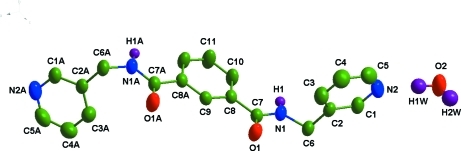
The molecular structure of the title compound with displacement ellipsoids drawn at the 50% probability level (symmetry code; (A) -x+1, y, -z+1/2). Only the symmetry unique water molecule is shown.

**Fig. 2.**
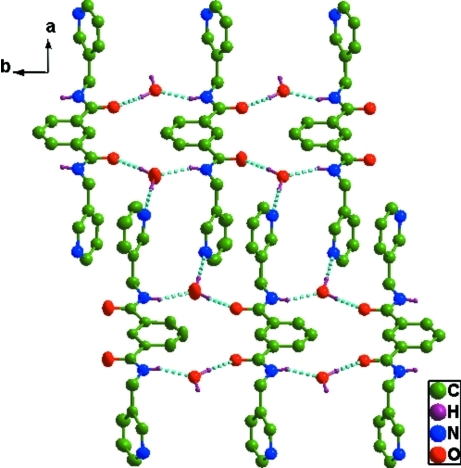
Part of the crystal structure with hydrogen bonds shown as dashed lines. Only H atoms involved in hydrogen bonds are shown.

## supplementary crystallographic information

### Comment

Amides are useful to construct long ligands for building porous metal-organic frameworks (Luo *et al.*, 2007;2009). We synthesized the title compound in the hope of using it as a ligand for constructing metal-organic frameworks. The crystal structure of the title compound is presented herein.

The molecular structure of the title compound is shown in Fig. 1. The molecule lies on a twofold rotation axis. The symmetry unique pyridine ring forms a dihedral angle of 83.16 (8)° with the central benzene ring. In the crystal, intermolecular N—H···O, O—H···N and O—H···O hydrogen bonds connect the components of the structure into a two-dimensional network parallel to (101) (Fig. 2).

### Experimental

Thionyl chloride (10 mL, 99.0%) and isophthalic acid (10 mmol) in a round bottomflask was refluxed for 2 h. After the reaction was complete, dichloromethane (30 mL), triethylamine (4.2 mL) and pyridin-3-ylmethanamine (20 mmol) were added to the solution, and stired for 2 h in an ice bath. The mixture was refluxed for 3 hr. The solvent was evaporated *in vacuo* and the residue was washed with water. The title compound was dissolved in *N*,*N*-dimethylformamide and single crystals were obtained by slow evaporation.

### Refinement

H atoms bonded to C and N atoms were placed in calculated positions with C—H = 0.93 - 0.95Å, N—H = 0.86Å and included using a riding-model approximation with *U*_iso_(H) = 1.2U_eq_(C,N). H atoms bonded to O atoms were refined independently with isotropic displacement parameters.

### Crystal data

**Table:**

C_20_H_18_N_4_O_2_·2H_2_O	*F*(000) = 808
*M**_r_* = 382.42	*D*_x_ = 1.328 Mg m^−^^3^
Monoclinic, *C*2/*c*	Mo *K*α radiation, λ = 0.71073 Å
Hall symbol: -C 2yc	Cell parameters from 2484 reflections
*a* = 23.0097 (8) Å	θ = 3.1–27.3°
*b* = 7.0040 (2) Å	µ = 0.10 mm^−^^1^
*c* = 12.4483 (4) Å	*T* = 296 K
β = 107.493 (2)°	Block, colorless
*V* = 1913.39 (11) Å^3^	0.20 × 0.20 × 0.15 mm
*Z* = 4	

### Data collection

**Table:**

Bruker SMART CCD diffractometer	1687 independent reflections
Radiation source: fine-focus sealed tube	1350 reflections with *I* > 2σ(*I*)
graphite	*R*_int_ = 0.026
φ and ω scans	θ_max_ = 25.0°, θ_min_ = 1.9°
Absorption correction: multi-scan (SADADS; Sheldrick, 1996)	*h* = −27→27
*T*_min_ = 0.981, *T*_max_ = 0.986	*k* = −8→8
7105 measured reflections	*l* = −14→14

### Refinement

**Table:**

Refinement on *F*^2^	Primary atom site location: structure-invariant direct methods
Least-squares matrix: full	Secondary atom site location: difference Fourier map
*R*[*F*^2^ > 2σ(*F*^2^)] = 0.039	Hydrogen site location: inferred from neighbouring sites
*wR*(*F*^2^) = 0.113	H atoms treated by a mixture of independent and constrained refinement
*S* = 1.02	*w* = 1/[σ^2^(*F*_o_^2^) + (0.0574*P*)^2^ + 0.8895*P*] where *P* = (*F*_o_^2^ + 2*F*_c_^2^)/3
1687 reflections	(Δ/σ)_max_ < 0.001
137 parameters	Δρ_max_ = 0.17 e Å^−^^3^
0 restraints	Δρ_min_ = −0.14 e Å^−^^3^

### Special details

**Table:**

Geometry. All e.s.d.'s (except the e.s.d. in the dihedral angle between two l.s. planes) are estimated using the full covariance matrix. The cell e.s.d.'s are taken into account individually in the estimation of e.s.d.'s in distances, angles and torsion angles; correlations between e.s.d.'s in cell parameters are only used when they are defined by crystal symmetry. An approximate (isotropic) treatment of cell e.s.d.'s is used for estimating e.s.d.'s involving l.s. planes.
Refinement. Refinement of *F*^2^ against ALL reflections. The weighted *R*-factor *wR* and goodness of fit *S* are based on *F*^2^, conventional *R*-factors *R* are based on *F*, with *F* set to zero for negative *F*^2^. The threshold expression of *F*^2^ > σ(*F*^2^) is used only for calculating *R*-factors(gt) *etc*. and is not relevant to the choice of reflections for refinement. *R*-factors based on *F*^2^ are statistically about twice as large as those based on *F*, and *R*- factors based on ALL data will be even larger.

### Fractional atomic coordinates and isotropic or equivalent isotropic displacement parameters (Å^2^)

**Table:**

	*x*	*y*	*z*	*U*_iso_*/*U*_eq_	
C1	0.26370 (7)	0.5013 (2)	0.48897 (15)	0.0532 (5)	
H1	0.2776	0.5491	0.5619	0.064*	
C2	0.30590 (7)	0.4263 (2)	0.44286 (13)	0.0440 (4)	
C3	0.28433 (8)	0.3535 (3)	0.33491 (15)	0.0614 (5)	
H3	0.3112	0.3006	0.3004	0.074*	
C4	0.22323 (9)	0.3594 (3)	0.27895 (16)	0.0706 (6)	
H4	0.2081	0.3095	0.2067	0.085*	
C5	0.18480 (8)	0.4404 (3)	0.33149 (19)	0.0681 (6)	
H5	0.1435	0.4469	0.2924	0.082*	
C6	0.37282 (7)	0.4242 (3)	0.50748 (13)	0.0500 (4)	
H6A	0.3792	0.4938	0.5774	0.060*	
H6B	0.3858	0.2933	0.5262	0.060*	
C7	0.43779 (6)	0.4081 (2)	0.38248 (13)	0.0442 (4)	
C8	0.47085 (6)	0.5210 (2)	0.31622 (12)	0.0402 (4)	
C9	0.5000	0.4233 (3)	0.2500	0.0402 (5)	
H9	0.5000	0.2905	0.2500	0.048*	
C10	0.47222 (7)	0.7191 (3)	0.31698 (14)	0.0544 (5)	
H10	0.4542	0.7863	0.3631	0.065*	
C11	0.5000	0.8164 (4)	0.2500	0.0655 (8)	
H11	0.5000	0.9492	0.2500	0.079*	
H1W	0.1383 (14)	0.563 (4)	0.494 (2)	0.126 (10)*	
H2W	0.0893 (14)	0.482 (5)	0.548 (3)	0.141 (11)*	
N1	0.40984 (5)	0.5090 (2)	0.44382 (11)	0.0470 (4)	
H1A	0.4140	0.6311	0.4457	0.056*	
N2	0.20366 (7)	0.5099 (2)	0.43535 (15)	0.0662 (5)	
O1	0.43603 (5)	0.23240 (18)	0.37774 (11)	0.0644 (4)	
O2	0.10649 (7)	0.5921 (2)	0.52588 (15)	0.0810 (5)	

### Atomic displacement parameters (Å^2^)

**Table:**

	*U*^11^	*U*^22^	*U*^33^	*U*^12^	*U*^13^	*U*^23^
C1	0.0526 (10)	0.0520 (10)	0.0644 (11)	−0.0028 (8)	0.0316 (8)	−0.0020 (8)
C2	0.0488 (8)	0.0400 (9)	0.0508 (9)	−0.0032 (7)	0.0263 (7)	0.0027 (7)
C3	0.0595 (10)	0.0687 (13)	0.0624 (11)	−0.0019 (9)	0.0282 (9)	−0.0078 (9)
C4	0.0648 (12)	0.0808 (15)	0.0648 (12)	−0.0124 (10)	0.0173 (10)	−0.0058 (11)
C5	0.0477 (10)	0.0721 (13)	0.0825 (14)	−0.0080 (9)	0.0167 (9)	0.0105 (11)
C6	0.0495 (9)	0.0574 (11)	0.0509 (9)	0.0000 (8)	0.0271 (7)	0.0031 (8)
C7	0.0406 (8)	0.0453 (10)	0.0512 (9)	0.0011 (7)	0.0205 (7)	0.0030 (7)
C8	0.0335 (7)	0.0442 (9)	0.0459 (8)	0.0006 (6)	0.0163 (6)	0.0010 (7)
C9	0.0361 (10)	0.0374 (11)	0.0496 (12)	0.000	0.0167 (9)	0.000
C10	0.0614 (10)	0.0458 (10)	0.0700 (11)	0.0029 (8)	0.0410 (9)	−0.0033 (8)
C11	0.0823 (18)	0.0390 (13)	0.097 (2)	0.000	0.0606 (16)	0.000
N1	0.0461 (7)	0.0473 (8)	0.0568 (8)	−0.0013 (6)	0.0295 (6)	0.0014 (6)
N2	0.0513 (9)	0.0639 (10)	0.0945 (13)	0.0020 (7)	0.0388 (8)	0.0039 (9)
O1	0.0800 (9)	0.0451 (8)	0.0886 (9)	0.0000 (6)	0.0566 (7)	0.0049 (6)
O2	0.0929 (10)	0.0548 (9)	0.1232 (13)	0.0019 (7)	0.0745 (10)	−0.0074 (8)

### Geometric parameters (Å, °)

**Table:**

C1—N2	1.343 (2)	C7—O1	1.232 (2)
C1—C2	1.372 (2)	C7—N1	1.3383 (19)
C1—H1	0.9300	C7—C8	1.504 (2)
C2—C3	1.383 (2)	C8—C10	1.388 (2)
C2—C6	1.508 (2)	C8—C9	1.3892 (17)
C3—C4	1.369 (3)	C9—C8^i^	1.3892 (17)
C3—H3	0.9300	C9—H9	0.9300
C4—C5	1.371 (3)	C10—C11	1.374 (2)
C4—H4	0.9300	C10—H10	0.9300
C5—N2	1.326 (3)	C11—C10^i^	1.374 (2)
C5—H5	0.9300	C11—H11	0.9300
C6—N1	1.4534 (18)	N1—H1A	0.8600
C6—H6A	0.9700	O2—H1W	0.95 (3)
C6—H6B	0.9700	O2—H2W	0.95 (3)
			
N2—C1—C2	124.12 (17)	O1—C7—N1	122.70 (14)
N2—C1—H1	117.9	O1—C7—C8	120.90 (14)
C2—C1—H1	117.9	N1—C7—C8	116.38 (14)
C1—C2—C3	117.08 (15)	C10—C8—C9	118.85 (14)
C1—C2—C6	121.21 (14)	C10—C8—C7	122.41 (13)
C3—C2—C6	121.70 (14)	C9—C8—C7	118.72 (14)
C4—C3—C2	119.80 (17)	C8^i^—C9—C8	121.0 (2)
C4—C3—H3	120.1	C8^i^—C9—H9	119.5
C2—C3—H3	120.1	C8—C9—H9	119.5
C3—C4—C5	118.73 (18)	C11—C10—C8	120.36 (15)
C3—C4—H4	120.6	C11—C10—H10	119.8
C5—C4—H4	120.6	C8—C10—H10	119.8
N2—C5—C4	123.23 (17)	C10^i^—C11—C10	120.5 (2)
N2—C5—H5	118.4	C10^i^—C11—H11	119.7
C4—C5—H5	118.4	C10—C11—H11	119.7
N1—C6—C2	112.14 (12)	C7—N1—C6	123.79 (14)
N1—C6—H6A	109.2	C7—N1—H1A	118.1
C2—C6—H6A	109.2	C6—N1—H1A	118.1
N1—C6—H6B	109.2	C5—N2—C1	117.01 (15)
C2—C6—H6B	109.2	H1W—O2—H2W	113 (2)
H6A—C6—H6B	107.9		
			
N2—C1—C2—C3	−0.9 (3)	N1—C7—C8—C9	−179.21 (11)
N2—C1—C2—C6	179.05 (15)	C10—C8—C9—C8^i^	−1.20 (11)
C1—C2—C3—C4	0.4 (3)	C7—C8—C9—C8^i^	177.47 (14)
C6—C2—C3—C4	−179.58 (17)	C9—C8—C10—C11	2.4 (2)
C2—C3—C4—C5	0.8 (3)	C7—C8—C10—C11	−176.19 (12)
C3—C4—C5—N2	−1.6 (3)	C8—C10—C11—C10^i^	−1.24 (11)
C1—C2—C6—N1	−127.37 (16)	O1—C7—N1—C6	−2.4 (2)
C3—C2—C6—N1	52.6 (2)	C8—C7—N1—C6	176.18 (13)
O1—C7—C8—C10	178.05 (16)	C2—C6—N1—C7	−95.97 (18)
N1—C7—C8—C10	−0.6 (2)	C4—C5—N2—C1	1.1 (3)
O1—C7—C8—C9	−0.6 (2)	C2—C1—N2—C5	0.2 (3)

Symmetry codes: (i) −*x*+1, *y*, −*z*+1/2.

### Hydrogen-bond geometry (Å, °)

**Table:**

*D*—H···*A*	*D*—H	H···*A*	*D*···*A*	*D*—H···*A*
N1—H1A···O2^ii^	0.86	2.05	2.859 (2)	156.
O2—H2W···O1^iii^	0.95 (3)	1.94 (3)	2.875 (2)	169 (3)
O2—H1W···N2	0.95 (3)	1.90 (3)	2.849 (2)	178 (3)

Symmetry codes: (ii) −*x*+1/2, −*y*+3/2, −*z*+1; (iii) −*x*+1/2, −*y*+1/2, −*z*+1.
